# Pomegranate (*Punica granatum* L.) in Veterinary Medicine: A Comprehensive Review of Pharmacological Activities and Species-Specific Therapeutic Applications

**DOI:** 10.3390/vetsci13080832

**Published:** 2026-08-19

**Authors:** Roberto Bava, Stefano Ruga, Giovanna Liguori, Antonio Giordano, Giancarlo Statti, Mariangela Marrelli, Vincenzo Musella, Ernesto Palma, Domenico Britti, Carmine Lupia, Fabio Castagna

**Affiliations:** 1 Department of Health Sciences, University “Magna Graecia” of Catanzaro, 88100 Catanzaro, Italy; roberto.bava@unicz.it (R.B.); rugast1@gmail.com (S.R.); musella@unicz.it (V.M.); palma@unicz.it (E.P.); britti@unicz.it (D.B.); fabiocastagna@unicz.it (F.C.); 2 Local Health Authority (ASL) Foggia, 71121 Foggia, Italy; 3 Sbarro Institute for Cancer Research and Molecular Medicine, Center for Biotechnology, Department of Biology, College of Science and Technology, Temple University, Philadelphia, PA 19122, USA; antonio.giordano@temple.edu; 4 Department of Pharmacy, Health and Nutritional Sciences, University of Calabria, 87036 Cosenza, Italy; giancarlo.statti@unical.it (G.S.); mariangela.marrelli@unical.it (M.M.); 5 Mediterranean Ethnobotanical Conservatory, 88054 Sersale, Italy; studiolupiacarmine@libero.it

**Keywords:** *Punica granatum*, pomegranate, phytobiotic, antiparasitic, antimicrobial, antioxidant, poultry, ruminants, veterinary pharmacology, immunomodulation, feed additive

## Abstract

*Punica granatum* L. (pomegranate) is one of the oldest medicinal plants known to humankind, valued across ancient civilisations for treating parasitic, microbial, and metabolic diseases. Its exceptionally rich phytochemical composition, dominated by punicalagins, ellagic acid, anthocyanins, flavonoids, piperidine alkaloids, and the unique conjugated fatty acid punicic acid, confers a remarkably broad spectrum of biological activities of direct relevance to contemporary veterinary medicine. While human-health applications have been extensively reviewed, a comprehensive synthesis of veterinary evidence across multiple species remains lacking. This review consolidates current preclinical and field knowledge on the pharmacological effects of pomegranate preparations in poultry, ruminants, swine, fish, companion animals, and laboratory models. In poultry—the most extensively studied taxon—dietary inclusion of pomegranate peel powder or extract consistently enhances growth performance, antioxidant status, and humoral immunity while exerting meaningful anticoccidial activity. The antiparasitic properties are compellingly supported by evidence against gastrointestinal nematodes of ruminants, tapeworms, schistosomes, and protozoa including *Giardia*, *Cryptosporidium*, and *Leishmania* spp., as well as monogenean fish parasites. Broad-spectrum antimicrobial activity extends to major veterinary pathogens such as *Salmonella*, *Escherichia coli*, *Staphylococcus aureus* (including MRSA), and *Clostridium perfringens*. Rodent models have validated antidiabetic, hepatoprotective, nephroprotective, and reproductive benefits, including improved post-thaw sperm quality and enhanced litter size. The safety profile is generally favourable at conventional doses, although high dietary inclusion elicits anti-nutritional effects from condensed tannins, and potential drug interactions via cytochrome P450 inhibition warrant clinical caution. Despite this substantial evidence, significant translational barriers persist, including extract heterogeneity, absence of pharmacokinetic data in target species, and scarcity of controlled clinical trials. By providing a species- and pathology-driven synthesis, this review identifies critical research priorities and highlights the immense potential of this ancient, accessible, and economically viable phytobiotic as a natural alternative to antibiotic growth promoters and synthetic antiparasitics in veterinary practice.

## Simple Summary

Pomegranate (*Punica granatum* L.) is one of the oldest medicinal plants known to humanity, used for thousands of years in traditional medicine systems. Today, its use in veterinary medicine is gaining increasing attention as farmers and veterinarians seek natural alternatives to antibiotics and synthetic drugs. This review examines the scientific evidence for using pomegranate preparations—particularly the peel, seed oil, and extracts—in various animal species including poultry, cattle, sheep, pigs, fish, dogs, and rabbits. We find that pomegranate products show considerable promise in treating parasitic infections, fighting harmful bacteria, reducing oxidative stress, and improving animal growth and reproduction. In poultry, pomegranate peel added to feed consistently improves growth, immunity, and resistance to coccidiosis. In ruminants, it shows activity against gastrointestinal worms. In fish, it rapidly kills certain external parasites. Despite this promising evidence, significant challenges remain, including variation in product quality, lack of standardised dosing, and insufficient clinical trials in target species. This review identifies key research priorities to translate these promising findings into practical veterinary applications.

## 1. Introduction

The *Punica granatum* L. (pomegranate, family Lythraceae, formerly Punicaceae) is one of the earliest cultivated fruit crops and medicinal plants in human history, with documented use dating to at least 3000 BCE in northern Iran, India, and the Mediterranean basin [1,2,3]. Its medicinal use spans Ayurvedic, Egyptian, Persian, Greek, and Chinese systems of medicine, where the fruit, rind, root, bark, flowers, and leaves have been prescribed for a remarkable range of conditions, from intestinal parasitoses and dysentery to cardiovascular disease and cancer [4,5].

Pomegranate is of particular interest for veterinary medicine because it combines, within a single, inexpensive, and widely available plant, antiparasitic, antimicrobial, antioxidant, and immunomodulatory properties that directly address some of the most pressing challenges currently facing the sector: antimicrobial and anthelmintic resistance, consumer demand for residue-free animal products, and the search for sustainable alternatives to synthetic growth promoters [6]. In the contemporary era of antimicrobial resistance, the ban on antibiotic growth promoters in livestock (EU, 2006) [7], along with growing consumer demand for residue-free animal products, has led to intense scientific and commercial interest on phytobiotics [8]. *P. granatum*, whose by-products—peel powder (PPP), peel extract (PPE), seed oil (PSO), pomace, and juice—represent inexpensive, abundant processing residues, retains or even exceeds the bioactivity of the intact fruit [9,10,11].

Despite an exponentially growing body of evidence in human medicine, comprehensive synthesis of veterinary applications remains sparse. Most available reviews focus primarily on single animal species or specific pharmacological activities [2]. To the best of our knowledge, no previous review has comprehensively integrated the full spectrum of animal species, from food animals (poultry, ruminants, swine) to companion animals, aquaculture species, and laboratory models, within a clinically oriented and species-specific framework.

The objective of this narrative review is to: (1) describe the phytochemistry of *P. granatum* with particular reference to the bioactive compounds relevant to veterinary pharmacology; (2) critically evaluate the evidence for species-specific applications across major veterinary disciplines; (3) analyse mechanisms of action at the cellular and molecular level; and (4) identify research gaps and translational priorities for clinical veterinary use.

## 2. Review Methodology

This comprehensive narrative review synthesises the current scientific evidence on the veterinary applications of *Punica granatum* L. (pomegranate, hereafter PG). A literature search was performed in PubMed, Scopus, and Google Scholar for articles published between 1990 and June 2026. Only articles published in English were considered. The search strategy combined Medical Subject Headings (MeSH) and free-text terms where appropriate, adapting syntax to the requirements of each database. The search combined the terms “*Punica granatum*”, “pomegranate”, “pomegranate peel extract”, and “pomegranate seed oil” with veterinary-specific keywords (poultry, broiler, ruminant, sheep, cattle, swine, fish, salmon, tilapia, catfish, aquatic species, and fish disease, dog, cat, rabbit, antiparasitic, antimicrobial, antioxidant, etc.).

Additional relevant studies were identified through manual screening of reference lists from key articles and previous reviews. Duplicate records were identified and removed before full-text evaluation. All original research articles, in vitro and in vivo studies, and reviews reporting data on the use of pomegranate preparations in non-human animal species were considered. Studies conducted exclusively in humans or focused solely on phytochemical analysis without biological evaluation were excluded. Conference abstracts, editorials, unpublished data, and studies lacking sufficient methodological details were also excluded.

Because of the marked heterogeneity of animal species, experimental models, pomegranate preparations, dosages, and outcome measures, quantitative meta-analysis was considered inappropriate. Therefore, findings were synthesized narratively and organized according to animal species and therapeutic application. Emphasis was placed on studies with potential translational relevance to clinical veterinary practice and livestock production. Acknowledging the breadth of the topic, this review adopts a species- and pathology-driven organizational framework to facilitate clinical translation for veterinary practitioners. For the preparation of figures, the layout and colour schemes of images initially drafted in Microsoft PowerPoint were refined using ChatGPT (version GPT-4, OpenAI). The use of this tool was limited exclusively to non-scientific aspects of visual presentation.

## 3. Phytochemical Profile of *Punica granatum*

The remarkable pharmacological versatility of *P. granatum* is primarily attributable to its chemically diverse phytocomplex, which comprises polyphenols, alkaloids, fatty acids, organic acids, vitamins, and minerals distributed unevenly across the different anatomical parts of the plant [11]. Understanding this phytochemical composition is essential for interpreting the species-specific biological activities discussed throughout this review. The biological activities of *P. granatum* are attributable to a complex, synergistic phytochemical matrix of polyphenols, alkaloids, fatty acids, and essential micronutrients distributed across different parts of the plant. Phytochemical composition varies with cultivar, geographic origin, growth conditions, processing method, and storage, which likely contributes to the variability observed among experimental studies [12].

### 3.1. Polyphenols

Punicalagins A and B (C48H28O30, MW 1084.7 g/mol) are the most abundant and structurally unique ellagitannins in pomegranate, reported to account for a substantial proportion of the antioxidant capacity of pomegranate juice [13]. They hydrolyse to ellagic acid and gallagic acid in the gastrointestinal tract, giving rise to urolithins, gut-microbiota-derived metabolites with potent anti-inflammatory and anti-proliferative properties [14]. Ellagic acid (C14H6O8, MW 302.19 g/mol) is a cellular antioxidant that activates the Nrf2/ARE pathway, suppresses NF-κB-driven inflammatory signalling [15].

Flavonoids, including quercetin, rutin, kaempferol, luteolin, apigenin, and catechins, and anthocyanins (delphinidin, cyanidin, pelargonidin), contribute to anti-inflammatory, antimicrobial, and antitumour properties [16]. Chlorogenic acid and coumaric acid are present in the peel and possess ACE-inhibitory and antihypertensive activity [9,17]. Beyond the polyphenolic fraction, pomegranate peel and pulp also contain structurally complex polysaccharides, notably pectic polysaccharides and arabinogalactans, which contribute to the prebiotic potential of pomegranate by-products by selectively promoting beneficial gut taxa such as *Lactobacillus* and *Bifidobacterium* [18], and which have themselves been reported to exert direct immunomodulatory activity through activation of macrophages and stimulation of cytokine release, independent of the polyphenolic constituents [19]. These polysaccharide fractions remain comparatively understudied relative to the polyphenolic components and represent a further avenue for veterinary application, particularly as prebiotic feed additives. The distribution of these major phytochemicals across the different anatomical parts of the plant and their primary veterinary biological functions are summarized in Table 1.

### 3.2. Alkaloids

Piperidine alkaloids, pelletierine, pseudopelletierine, methylisopelletierine, and methylpelletierine, are concentrated in the root bark and peel [20]. Pelletierine (C8H15ON) and related compounds are the pharmacological basis of the classical taeniacidal and vermifugal use of pomegranate root bark, acting by inducing spastic paralysis of intestinal cestodes and nematodes [2,6,21].

### 3.3. Fatty Acids and Lipid Fractions

**Figure 1:**
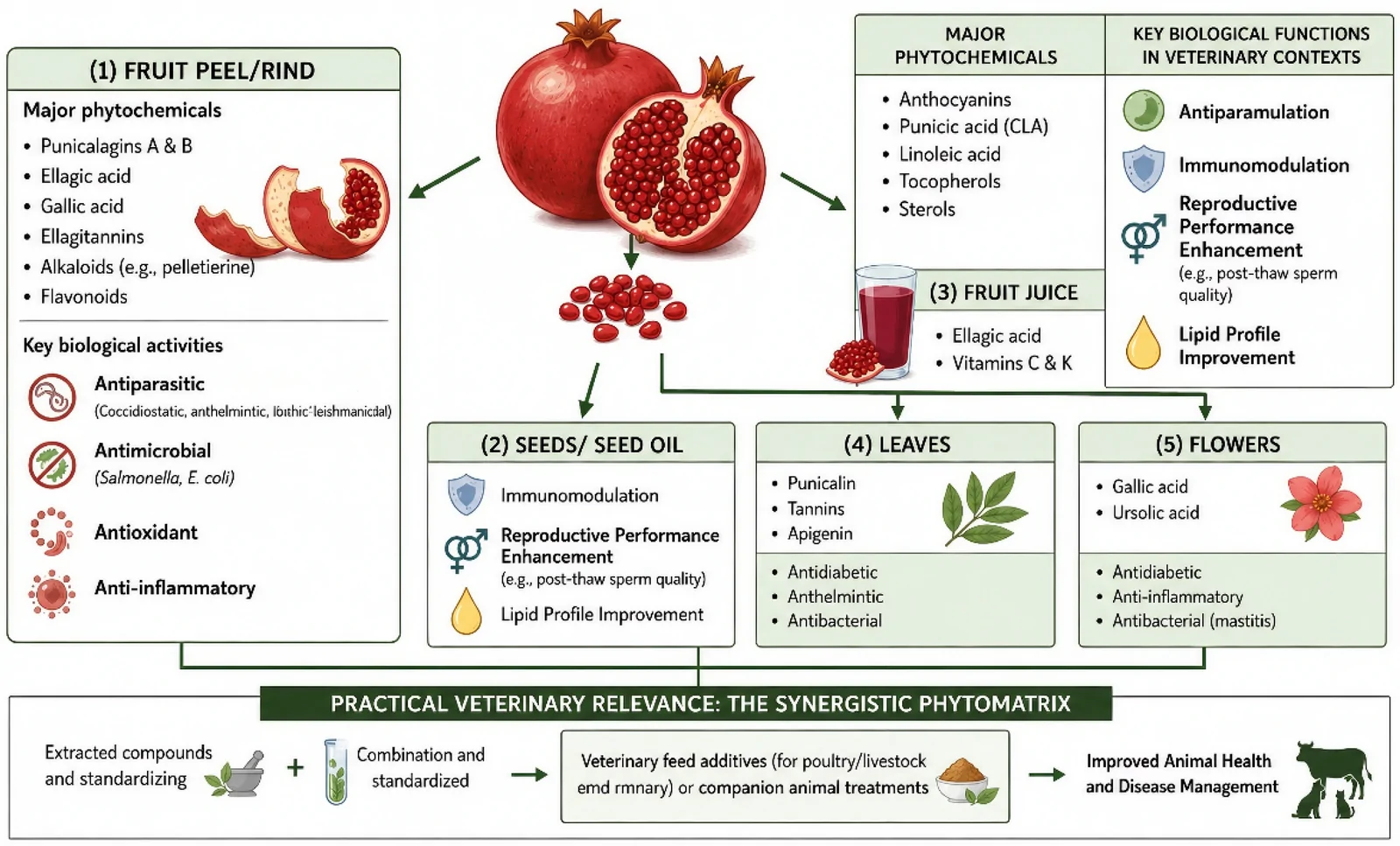
The figure provides an overview of the distribution of the principal phytochemicals across the anatomical parts of *P. granatum* and summarizes their main biological functions relevant to veterinary medicine.

Pomegranate seed oil is exceptionally rich (70–80% of total fatty acids) in punicic acid (cis-9, trans-11, cis-13-octadecatrienoic acid), a conjugated linolenic acid isomer unique to pomegranate among food sources [22]. Punicic acid exerts immunomodulatory activity, modulating lymphocyte subset ratios and reducing pro-inflammatory cytokine production [23], and has been shown to improve peripheral insulin sensitivity in obese animal models [5,10,24]. A visual synthesis of the phytochemical distribution across the different anatomical parts of *P. granatum* and their corresponding veterinary biological functions is presented in Figure 1, which maps each plant part to its major constituents and key pharmacological activities discussed throughout this section.

The peel/rind constitutes approximately 40–50% of total fruit weight and represents the primary industrial by-product of juice extraction. Its extraordinarily high polyphenol content, approximately 10× that of the pulp [25], makes PPE and PPP the most pharmacologically active and practically relevant fractions for veterinary applications. From a sustainability perspective, the valorization of peel by-products is also consistent with current circular economy strategies aimed at reducing agro-industrial waste while generating high-value veterinary nutraceuticals.

The biological activity of pomegranate preparations depends not only on the plant part used but also on extraction procedures, solvent composition, processing conditions, and phytochemical standardization [26]. Variability in extraction methodology represents one of the principal factors contributing to differences in biological efficacy among published studies. Consequently, understanding extraction technologies and their influence on phytochemical yield is essential for interpreting experimental findings and for future clinical standardization.

### 3.4. Phytochemical Extraction Methods (Traditional and Novel)

The biological efficacy of *P. granatum* extracts is critically dependent on the extraction method employed. Traditional methods, including cold maceration, decoction, percolation, and Soxhlet extraction, are simple and cost-effective but present limitations such as long extraction times, high solvent consumption, and potential degradation of thermolabile compounds [27,28,29]. Decoction, for instance, promotes cell wall disruption but may degrade sensitive polyphenols [27], while Soxhlet extraction, although highly efficient, risks thermal degradation of bioactive constituents [30,31,32].

Emerging “green” technologies offer significant advantages over conventional methods. Ultrasound-assisted extraction (UAE) utilizes acoustic cavitation to break cell walls, facilitating compound release [33]. For pomegranate extracts, UAE under optimized conditions (15–40 min, 30–50 °C, 75–500 W, ethanol/water solvents) increases the yield of punicalagins and ellagic acid by 30–50% compared to maceration [34,35]. Microwave-assisted extraction (MAE) employs dielectric heating, achieving high extraction efficiencies in minutes (2–10 min, 400–800 W, 50–80 °C) with minimal temperature increase [36,37]. Supercritical fluid extraction (SFE) using supercritical CO_2_ is non-toxic and non-flammable, preserving compound stability; it has been successfully applied to extract ellagic acid and essential oils from pomegranate peel [34,38].

### 3.5. Critical Considerations on Pan-Assay Interference Compounds (PAINS)

In the evaluation of the biological activity of polyphenol-rich plant extracts such as those from *P. granatum*, it is imperative to consider the phenomenon of “Pan-Assay Interference Compounds” (PAINS) [39,40]. Polyphenols, particularly ellagitannins and flavonoids, possess structural features (e.g., ortho-diphenolic groups, multiple hydroxyl groups) that can generate false positives in in vitro bioassays through non-specific interference mechanisms, rather than genuine interaction with the molecular target of interest [41].

The principal mechanisms of interference include: (1) non-specific chemical reactivity—ortho-diphenol groups can oxidize to electrophilic quinones that covalently react with protein thiol groups; (2) formation of colloidal aggregates—hydrolyzable tannins such as punicalagin can form supramolecular aggregates that non-specifically sequester proteins; (3) interference with detection systems—many polyphenols absorb in the UV-Vis range or are fluorescent, interfering with absorbance-based assays (e.g., MTT, NADH) and fluorescence-based assays; (4) metal ion chelation—the arrangement of hydroxyl groups allows chelation of metals such as iron or copper, which can interfere in metal-dependent assays; and (5) redox interference—polyphenols can generate reactive oxygen species or act as antioxidants in cellular assays, altering the redox state and generating non-specific signals in viability or inflammation assays [39,40].

Although these properties represent an interpretative challenge, they do not invalidate the therapeutic potential of the phytocomplex. First, PAINS artifacts are largely assay-dependent [42]; in more complex biological systems (cell-based assays, tissue models, or in vivo settings), non-specific interference is often mitigated by serum proteins, membrane barriers, or cellular metabolism [42,43]. Second, numerous approved drugs or clinical candidates (e.g., quercetin, epigallocatechin gallate) exhibit PAINS properties in vitro yet manifest genuine pharmacological effects in living organisms [44]. Third, polyphenols from *P. granatum* can act through mechanisms that bypass classical enzyme inhibition, such as modulation of transcription factors (NF-κB, Nrf2), gut microbiota conversion into bioactive metabolites (e.g., urolithins), or synergistic interactions within the whole extract [45,46]. Finally, the application of orthogonal validation methods (detergent controls, reducing agents, metal chelators, non-optical detection techniques) allows researchers to discriminate between artifactual interference and true biological activity [47]. Therefore, while PAINS flags should prompt rigorous experimental scrutiny, they should not be used as an outright exclusion criterion for natural products derived from *P. granatum*.

## 4. Applications in Poultry Production

Among all veterinary fields, poultry production currently provides the most extensive body of evidence supporting the use of *P. granatum*-derived products. This reflects the growing interest in phytogenic feed additives [48] as sustainable alternatives to antibiotic growth promoters and synthetic anticoccidial agents [49] following increasingly restrictive regulatory policies. The progressive ban on antibiotic growth promoters since 2006 [7], combined with the high cost of coccidiostats and growing public concern about drug residues in poultry products [50], has driven intensive research into phytobiotic alternatives. PG by-products have been studied across broiler chickens, laying hens, Japanese quail, and other avian species, encompassing growth performance, carcass quality, antioxidant status, immune function, blood biochemistry, antimicrobial activity, and antiparasitic efficacy [6,48].

### 4.1. Growth Performance and Feed Efficiency in Broilers

The growth-promoting potential of PPP and PPE in broilers is well documented, although outcomes depend critically on dose, composition of the base diet, and form of the supplement. Multiple controlled trials with PPP at 0.01–1.50% of diet demonstrate improvements in body weight (BW), body weight gain (BWG), feed intake (FI), and feed conversion ratio (FCR). Elnaggar et al. (2022) [51] reported that dietary PPP at 0.25–1.50% improved productive performance, carcass traits, and protein profile in broilers, accompanied by increased weights of the spleen, thymus, and bursa of Fabricius. Hamady et al. (2015) [52] demonstrated that 0.01% PPE significantly enhanced feed intake, body weight gain, and dressed weight percentage in broiler chickens.

The mechanistic basis for growth promotion includes: (1) inhibition of pathogenic intestinal bacteria by phenolic acids and tannins, enhancing the lactic acid bacteria-to-pathogen ratio [53]; (2) inhibition of pancreatic lipase activity by PPE, modulating intestinal fat absorption and improving energy balance [54]; (3) proanthocyanidins increasing digestive enzyme activity and villus length-to-crypt depth ratio, thereby expanding absorptive surface area [55]; and (4) reduction in intestinal crypt depth, promoting mucosal renewal [56,57,58,59].

Conversely, high dietary inclusion levels (≥8%) consistently reduce growth performance, attributable to anti-nutritional effects of condensed tannins: inhibition of digestive enzymes (trypsin, amylase, lipase) by protein–polyphenol complexation, reduction in nitrogen balance and amino acid digestibility, and interference with calcium and fat absorption [60,61]. This dose-dependent duality underscores the importance of optimising supplementation levels. This finding emphasizes the importance of standardizing both the formulation and the inclusion rate of pomegranate-derived products before large-scale commercial implementation.

### 4.2. Carcass Traits and Meat Quality

Dietary supplementation with PPP and PPE consistently improves broiler carcass quality by reducing lipid oxidation in breast and thigh muscles, extending refrigerated shelf life, and modifying fatty acid profiles [62]. Pomegranate seed oil supplementation significantly increases polyunsaturated fatty acid and conjugated linoleic acid content in breast meat lipids, a desirable nutritional profile [63]. These changes may also enhance consumer acceptance by improving the nutritional quality and oxidative stability of poultry meat [64]. PPE at up to 300 mg/kg significantly increased total phenol and flavonoid content and antioxidant activity in fresh breast meat [65]. Abdel Baset et al. (2022) [66] demonstrated that PPP reduced the liver-to-carcass weight ratio, suggesting hepatoprotection. High inclusion of PPP (8%) decreased breast muscle crypt depth and the villus/crypt ratio, indicating a need for dose optimisation [66].

### 4.3. Antioxidant Activity in Poultry

The antioxidant capacity of pomegranate by-products in poultry is among the most consistently documented effects [48]. Mechanistically, following intestinal absorption, dietary polyphenols enhance systemic antioxidant defences, scavenging-free radicals, inhibiting lipid peroxidation, and upregulating antioxidant enzymes including catalase (CAT), superoxide dismutase (SOD), and glutathione peroxidase (GPx) [59]. These molecular actions translate into systemic effects, as serum malondialdehyde (MDA)—one of the most used biomarkers of lipid peroxidation and reactive oxygen species (ROS) damage—is consistently reduced in broilers and layers fed PPP or PPE [59]. At the production level, this antioxidant activity translates into tangible improvements in meat quality: PPE demonstrates efficacy comparable to α-tocopherol in preventing oxidative rancidity in refrigerated broiler meat, a clinically meaningful benchmark given the economic importance of lipid oxidation in chilled poultry products [67]. Furthermore, in heat-stressed broilers—a major welfare and productivity concern in tropical and subtropical systems—PPE at 250–650 mg/kg significantly decreased breast muscle MDA, an effect associated with improved water-holding capacity during storage and reduced incidence of pale-soft-exudative (PSE)-like lesions in the same study [68].

### 4.4. Immunomodulation

Pomegranate by-products exert significant immunomodulatory effects in poultry, acting through multiple mechanisms [48]. Dietary PPP increases the weight of lymphoid organs (spleen, bursa of Fabricius, thymus) [69], elevates serum concentrations of IgA, IgG, and IgM, and enhances antibody titres against Newcastle disease virus (NDV) vaccine [48,51]. The polyunsaturated fatty acid content of pomegranate seeds, particularly α-linolenic and linoleic acids, stimulates lymphocyte proliferation and increases immune organ weight [69].

The combinatorial effect of *P. granatum* and *Terminalia chebula* (PgTc) extracts in chickens challenged with avian pathogenic *Escherichia coli* (APEC) deserves specific mention: PgTc at 0.15–0.6 g/kg BW for seven days significantly increased survival rate and modulated immunity by reversing the overexpression of Toll-like receptors (TLR2, TLR4, TLR5) and inhibiting pro-inflammatory cytokines (IL-8, IL-1β, TNF-α, VCAM-1, ICAM-1) [70]. This mechanistic insight is particularly valuable given the global spread of drug-resistant APEC strains.

### 4.5. Antimicrobial Effects in Poultry

The antibacterial activity of PPP and PPE against Gram-positive and Gram-negative bacteria pathogenic to poultry has been confirmed in multiple in vitro and in vivo studies. Ahmed & Yang (2017) demonstrated that PPP at 0.5–1.0% significantly increased intestinal *Bacillus* spp. and *Saccharomyces cerevisiae* counts while reducing *E. coli* and *Salmonella* spp. counts in broiler excreta, with concomitant reduction in odorous gas (NH_3_, H2_S_) emissions [71]. This latter effect has direct implications for environmental management and worker welfare in commercial poultry facilities.

Against *Clostridium perfringens*, the causative agent of necrotic enteritis, a disease causing annual global losses exceeding USD 6 billion in poultry production, ellagic acid supplementation at 75 mg/kg diet ameliorated subclinical necrotic enteritis in broiler chickens by regulating jejunal inflammation signalling pathways, reducing intestinal oxidative stress, balancing cecal microbiota, and inhibiting intestinal barrier damage [72]. PPP at 5–10 g also reduced cecal *C. perfringens* count without impairing antioxidant enzyme activity [72].

### 4.6. Anticoccidial Activity

Coccidiosis caused by *Eimeria* spp. is arguably the most economically significant parasitic disease of commercial poultry, with global annual losses estimated at over USD 14 billion [73]. The emergence of ionophore-resistant *Eimeria* strains and regulatory restrictions on anticoccidials in organic and welfare-certified production systems create urgent demand for effective natural alternatives.

Pomegranate peel powder (PPP) has emerged as a promising phytogenic candidate, with recent evidence supporting its anticoccidial efficacy. In a well-controlled study by Hafeez et al. (2023) [74], 600 Ross 308 broilers were experimentally infected with *Eimeria tenella* (5 × 10^4^ sporulated oocysts/bird) and supplemented with PPP at 3 g/kg or 6 g/kg of feed, with amprolium (1 g/kg) serving as the reference anticoccidial treatment. The results demonstrated that PPP supplementation at 6 g/kg significantly improved growth performance in infected birds, with weight gain and feed conversion ratio approaching those of the amprolium-treated group. Importantly, both PPP doses significantly reduced cecal lesion scores and oocyst shedding (*p* < 0.05) compared to the infected untreated control, with the 6 g/kg dose showing superior efficacy. Histological examination revealed restoration of cecal villus architecture, including increased villus height, reduced crypt depth, and diminished inflammatory cell infiltration in PPP-supplemented birds.

The anticoccidial activity of PPP is attributed to its rich polyphenolic profile, particularly punicalagin, ellagitannins, and anthocyanins, which constitute the predominant bioactive compounds in pomegranate peel. These compounds are thought to exert antiprotozoal effects through disruption of parasite cell membrane integrity and inhibition of protozoal cell cycle progression, while also providing anti-inflammatory and antioxidant protection to intestinal epithelial cells. Notably, PPP offers the additional advantage of utilising an agricultural by-product, presenting a cost-effective and environmentally sustainable alternative to synthetic anticoccidials. These findings collectively support the potential of pomegranate peel powder as a natural anticoccidial agent, although further research is warranted to optimise dosage protocols and validate efficacy under commercial production conditions.

The specific mechanism involves punicalagin, the predominant active compound, which at pharmacological concentrations disrupts *Eimeria tenella* cell membrane integrity and inhibits protozoal cell cycle progression [75].

### 4.7. Egg Production and Quality in Layers and Quails

Dietary supplementation with pomegranate by-products has shown beneficial effects on laying hen performance. Supplementation with 4 g/kg pomegranate seed significantly increased the egg production rate, while dietary inclusion of 3 or 4 g/kg pomegranate seed tended to increase yolk colour [76]. Eid et al. (2021) [77] found that dietary pomegranate by-product alleviated dexamethasone-induced oxidative stress in laying hens during the pre-peak period, reducing MDA and enhancing antioxidative enzymes and total antioxidant blood capacity, a clinically relevant model of stress-related production decline. Pomegranate seed oil supplementation in laying hens produced dose-dependent increases in FI, egg production, and egg mass, attributed to antioxidant effects and improvement of intestinal enzymatic activity [78]. In Japanese quail, PPP at 4.5–7.5% increased egg production, egg weight, and egg mass without impairing growth performance [79]. Collectively, these findings indicate that pomegranate by-products may improve both productive efficiency and oxidative stability in laying birds without adversely affecting egg quality when administered within appropriate dietary ranges. The principal effects of pomegranate supplementation on production performance, antioxidant status, immune function, and metabolic parameters across poultry species are consolidated in Table 2.

## 5. Antiparasitic Activity: Mechanisms and Evidence Across Host Species

The antiparasitic properties of *P. granatum* represent one of its oldest and most consistently validated applications, with historical records from ancient Egypt (tapeworm treatment) and Ayurvedic medicine (“a pharmacy unto itself”) aligning with contemporary scientific evidence [2]. The primary active compounds responsible for antiparasitic activity are alkaloids (especially pelletierine), ellagitannins (punicalagin, ellagic acid), tannins, and flavonoids, acting via multiple complementary mechanisms [6]. The growing emergence of antiparasitic drug resistance in veterinary medicine has renewed interest in plant-derived compounds capable of acting through multiple complementary mechanisms, making pomegranate one of the most extensively investigated botanical candidates for integrated parasite control.

### 5.1. Mechanisms of Antiparasitic Action

**Figure 2:**
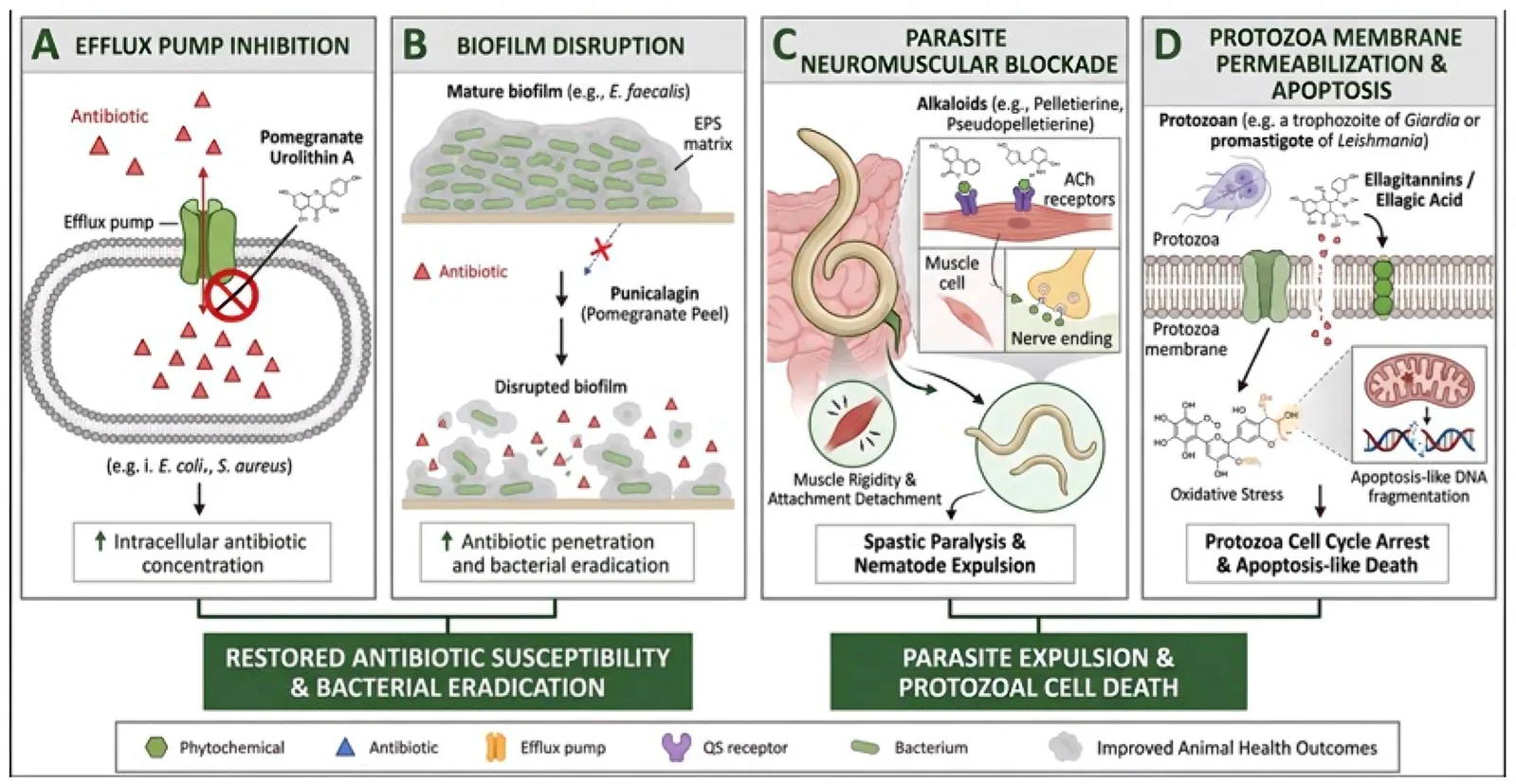
Dual Mechanisms of Action of Pomegranate: Antimicrobial and Antiparasitic Pathways. This figure illustrates four distinct cellular and molecular mechanisms of action underlying the therapeutic effects of pomegranate extracts. Panels (**A**,**B**) delineate how specific bioactive constituents (such as Urolithin A and Punicalagin) successfully inhibit bacterial efflux pumps and disrupt established biofilms, thereby restoring susceptibility and preventing microbial persistence. Panels (**C**,**D)** provide novel visualizations for this review: Panel (**C**) demonstrates the mechanism by which pomegranate alkaloids (e.g., pelletierine) induce neuromuscular blockade, leading to spastic paralysis in parasitic nematodes; Panel (**D)** illustrates how ellagitannins and ellagic acid trigger intracellular oxidative stress and induce an apoptosis-like programmed cell death pathway within protozoan parasites.

The antiparasitic activity of *P. granatum* is mediated through five complementary mechanisms affecting both parasite structures and host immune cells: (1) membrane disruption, whereby polyphenols interact with phospholipid bilayers and membrane proteins, altering permeability and inducing parasite lysis [21,81]; (2) alkaloid-mediated neuromuscular paralysis, in which pelletierine and related piperidines interfere with acetylcholine receptor signalling, causing spastic paralysis of helminth musculature, detachment from the intestinal wall, and expulsion [82]; (3) oxidative stress induction, as ellagic acid and punicalagin generate reactive oxygen species within protozoan cells (haemoflagellates and apicomplexans), overwhelming antioxidant defences and triggering apoptosis [21,83]; (4) metabolic enzyme inhibition, through which phenolic compounds impair parasite nucleotide synthesis and energy metabolism [84]; and (5) immunomodulation, whereby pomegranate compounds activate macrophages (enhancing pathogen clearance), modulate lymphocyte responses (humoral and cell-mediated immunity), and upregulate antioxidant enzymes in enterocytes and hepatocytes, thereby amplifying host clearance of parasitic burdens [85]. This multitarget action—directly affecting parasite membranes, neuromuscular function, and metabolic enzymes, while simultaneously mobilising host immune cells—reduces the likelihood of resistance development compared to single-target drugs, as schematically illustrated in Figure 2.

### 5.2. Anthelmintic Activity

Evidence for the anthelmintic activity of *P. granatum* spans all major helminth groups, including cestodes, trematodes, and nematodes, with efficacy demonstrated in both experimental models and naturally infected animals.

#### 5.2.1. Cestodes (Tapeworms)

The use of pomegranate root bark as a taeniacide pre-dates modern pharmacology. Pelletierine, pseudopelletierine, and methylpelletierine are the primary alkaloids responsible for this effect, loosening the cestode’s grip on the intestinal wall and facilitating expulsion [2,21]. In a key in vitro study, Al-Megrin (2016) demonstrated that pomegranate peel extract at 300 mg/kg reduced egg excretion and worm burden of *Hymenolepis nana* in experimentally infected Swiss albino mice, with the effect proportional to dose and treatment duration [86]. Abbas et al. (2020) confirmed anthelmintic activity of pomegranate peel extracts against *H. nana* in albino mice, with a substantial drop in viable egg count after seven days of treatment, showing efficacy approaching that observed with praziquantel under the experimental conditions investigated [87].

For *Hymenolepis nana*, the most prevalent human and rodent tapeworm worldwide, affecting up to 75 million individuals [2], these findings are particularly significant in the context of developing safe, low-cost alternatives to benzimidazoles in resource-limited settings.

#### 5.2.2. Trematodes (Flukes)

Pomegranate peel and leaf extracts exert potent antischistosomal activity, achieving 100% in vitro mortality of *Schistosoma mansoni* schistosomules and mature worms within 24 h [88], and 72–77% adult worm mortality in vivo following oral administration at 800 mg/kg for 45 days [89]. Ethanolic extracts induce motor paralysis, tegumental damage, and surface abnormalities [90], effects attributed to punicalagin and ellagic acid, which inhibit thioredoxin glutathione reductase, the parasite’s principal antioxidant enzyme [89]. Although schistosomiasis has limited veterinary relevance, these findings provide evidence supporting pomegranate’s broad antiparasitic potential.

#### 5.2.3. Nematodes (Roundworms)

PPE demonstrates dose-dependent anthelmintic activity against *Ascaris suum* (82% mortality at 75% concentration) [82], with efficacy also confirmed against gastrointestinal nematodes of ruminants [91,92,93,94,95]. In sheep, oral administration of whole-fruit macerate (50 mL/animal) reduced FEC by 50% at day 7 and 51% at day 21 [96]. A methanolic peel extract (200 mg/kg) achieved 85–97% FEC reduction across cattle, sheep, goats, and buffaloes, with efficacy approaching ivermectin and albendazole [97]. Activity correlates with phenolic acids (gallic, ellagic, syringic) and tannins and is broad-spectrum across nematode genera, though efficacy varies with preparation, parasite species, and dosage [21].

#### 5.2.4. Cestocidal Activity Against Echinococcus

Pomegranate flower extract exhibits concentration- and time-dependent protoscolicidal activity against *Echinococcus granulosus*, with LC50 values of 11.1 mg/mL at 15 min and 6.6 mg/mL at 60 min, and LT50 decreasing from 79.4 min (lowest concentration) to 18.3 min (highest) [98]. Notably, combining aqueous peel extract with albendazole enhances hydatid cyst inhibition and hepatoprotection compared to albendazole alone [99], effects attributed to punicalagin and other polyphenols. These findings suggest pomegranate extracts as potential adjuvant therapy for cystic echinococcosis, though clinical trials are warranted.

#### 5.2.5. Nematicidal Activity Against *Parascaris equorum*

*Parascaris equorum* is a common intestinal nematode of foals, causing significant gastrointestinal morbidity and mortality, with emerging resistance to macrocyclic lactones, pyrantel salts, and piperazine driving the search for alternatives [100]. Methanolic extracts of *P. granatum* flowers show significant concentration- and time-dependent larvicidal activity against *P. equorum* at 50–125 mg/mL [101]. Despite being less potent than *Capsicum annuum* in vitro, pomegranate flower extracts exhibit substantial activity, consistent with its broad anthelmintic effects against cestodes, nematodes, and protozoa [101]. These findings support pomegranate flower extracts as a promising component of integrated parasite management in equine medicine, warranting further in vivo evaluation in foals.

### 5.3. Antiprotozoal Activity

#### 5.3.1. Giardiasis

*Giardia lamblia*, the third most common cause of diarrhoeal disease worldwide, infects virtually all mammalian species. Al-Megrin (2017) reported a cure rate of 97.4% in *Giardia*-infected mice treated with PPE by day 28, with a significant reduction in cyst excretion from day 10 onward [86]. El-Kady et al. (2021) found that ethanolic PPE destroyed *G. lamblia* cysts in vitro (confirmed by eosin vital staining) and that the number of trophozoites and cysts in the small intestine of treated rats was significantly lower than in the metronidazole-positive control group, a striking result suggesting efficacy comparable to metronidazole under the experimental conditions evaluated [102].

The mechanisms proposed include membrane disruption by phenolic compounds, inhibition of cyst wall biogenesis by tannins, and antioxidant-mediated trophocyte killing [102]. Given the increasing prevalence of metronidazole-resistant *Giardia* in companion animals and livestock, these findings have direct clinical relevance.

#### 5.3.2. Cryptosporidiosis

*Cryptosporidium parvum* is a leading cause of profuse watery diarrhoea in neonatal calves, lambs, foals, and young pigs, as well as a significant cause of mortality in immunocompromised animals. Available treatments are largely ineffective (nitazoxanide has partial efficacy only) and supportive care remains the mainstay. Al-Mathal & Alsalem (2012) demonstrated in neonatal albino mice that aqueous PPE at 3 g/kg eliminated oocyst shedding completely, improved intestinal histology, and promoted continuous weight gain during infection, a result of considerable translational significance given the lack of effective veterinary interventions [103]. Given the limited therapeutic options currently available for cryptosporidiosis, these findings deserve further validation in naturally infected livestock species.

#### 5.3.3. Malaria (Plasmodium)

Although malaria is primarily a disease of human and primate importance, the avian malaria analogue caused by *Plasmodium relictum* affects wild and captive birds globally, and the rodent model of *P. falciparum* is among the most pharmacologically validated preclinical platforms. Dell’Agli et al. (2009) [104] demonstrated potent antiplasmodial activity of pomegranate fruit rind, identifying ellagic acid as the principal active compounds, with IC50 values in the low microgram range (2.8–4.5 μg/mL) against chloroquine-resistant *P. falciparum* strains. Ellagic acid and punicalagin suppress parasite growth by inhibiting plasmepsins and interfering with haemoglobin degradation and β-hematin formation [104]. Mubaraki et al. (2016) demonstrated in a murine malaria model that pomegranate rind extract reduced spleen injury, inflammatory and oxidative stress responses, and improved the innate immune response of the host, suggesting its potential as an alternative therapy for malaria [105].

#### 5.3.4. Leishmaniasis

Leishmania species infect dogs (*L. infantum*), causing visceral leishmaniasis and significant morbidity in Mediterranean, Middle Eastern, and Latin American canine populations [106]. Imperatori et al. (2019) [107] characterised by NMR and HPLC a pomegranate peel extract rich in punicalagin and demonstrated dose-dependent inhibition of *L. infantum* promastigote growth in vitro, with the PPE exhibiting apoptosis-mediated leishmanicidal activity confirmed by DNA fragmentation and real-time PCR. Morphological and ultrastructural changes were documented for the first time using electron microscopy [107]. Beyond the leishmanicidal effects already described, the study has deepened the mechanisms of action on *L. infantum*. Pomegranate peel extract, rich in hydrolyzable tannins (particularly α- and β-punicalagin), demonstrated dose-dependent antiproliferative activity with an IC50 (72 h) of 52.4 μg/mL. The most relevant findings included DNA degradation and ultrastructural alterations, particularly mitochondrial swelling, nuclear membrane rupture, and chromatin changes, as well as the appearance of vacuoles without content in the protozoa [107]. These findings confirm that the leishmanicidal effect is mediated through apoptosis-like cell death.

#### 5.3.5. Anti-Toxoplasma Activity

Toxoplasmosis, caused by *Toxoplasma gondii*, is a zoonotic infection of considerable veterinary relevance, affecting warm-blooded animals and causing severe brain complications, particularly in immunocompromised individuals, children, and pregnant women [108]. An aqueous extract of *P. granatum* peel demonstrated in vitro growth inhibition of *T. gondii* tachyzoites with an IC50 of 400–500 μg/mL, exhibiting low toxicity toward host cells (human foreskin fibroblasts) with a selectivity index of 2.4 [109]. Phytochemical analysis revealed that the extract contains high levels of phenols, gallic tannins, and sterols. Importantly, the extract induced apoptosis-like effects in *T. gondii* tachyzoites, evidenced by phosphatidylserine exposure on the tachyzoite surface and DNA fragmentation [109].

Furthermore, treatment with urolithin-A (UA), a gut-microbiota-derived metabolite produced from ellagitannins of pomegranate, reduced the number of tachyzoites and impaired cyst development (resulting in smaller cysts) in differentiated human neuronal cells in vitro. In a murine chronic toxoplasmosis model, UA administration confirmed interference with cyst maturation and notably attenuated the behavioural manipulation induced by the parasite, specifically restoring the natural aversion of infected animals to cat odour [110]. These findings suggest that pomegranate-derived compounds, particularly UA, may represent a promising strategy for managing neurotoxoplasmosis and its behavioural consequences. These observations also suggest that gut microbiota-derived metabolites such as urolithin A may contribute substantially to the in vivo efficacy of pomegranate beyond the activity of the native polyphenols themselves.

#### 5.3.6. Anti-Piroplasm Activity (Babesia and Theileria)

Piroplasmosis, caused by *Babesia* and *Theileria* species, are important tick-borne diseases affecting livestock and humans, leading to economic losses and health concerns worldwide [111]. The methanolic extract of *P. granatum* peel demonstrated notable inhibitory activity against various piroplasm parasites in vitro [112]. It significantly reduced the growth of *Theileria equi* with an IC50 of 100 ± 16.20 μg/mL and exhibited inhibitory activity against several *Babesia* species with IC50 values of 154.45 ± 23.11 μg/mL for *B. bovis*, 40.90 ± 9.35 μg/mL for *B. bigemina*, 72.71 ± 14.77 μg/mL for *B. divergens*, and 77.27 ± 16.94 μg/mL for *B. caballi* [112].

Notably, the combination of pomegranate peel extract with diminazene aceturate (an anti*Babesial* drug) on *B. bovis* revealed a synergistic or additive interaction, depending on the concentrations used [112,113]. In vivo, the combination therapy using lower doses of pomegranate extract in mice infected with *B. microti* considerably reduced parasitemia and mitigated the hemolytic anaemia associated with babesiosis [112]. Although *B. microti* was used here as an experimental rodent model, this finding has direct translational relevance for dogs, which are clinically important hosts for several Babesia species (*B. canis*, *B. gibsoni*, *B. vogeli*) and in which babesiosis can cause severe, potentially life-threatening haemolytic disease, particularly in immunocompromised patients; these results therefore support further evaluation of pomegranate-derived compounds as adjunctive antibabesial agents in canine practice [114,115]. The antipiroplasm activity is attributed to polyphenolic constituents, ellagitannins, flavonoids, and phenolic acids, which have been linked [116,117,118] to membrane disruption, oxidative stress induction, and interference with metabolic pathways essential for intraerythrocytic parasite replication. The synergistic interaction observed with diminazene aceturate suggests complementary mechanisms that may enhance antiparasitic efficacy and potentially reduce the required doses of synthetic drugs.

#### 5.3.7. Coccidiosis in Poultry

PPE at 400 ppm provides significant anticoccidial efficacy against *Eimeria* spp. in broilers [74]. The active principle punicalagin disrupts apicomplexan cell membrane integrity and inhibits cell cycle progression [49]. The parallel between this mechanism and the action of ionophore coccidiostats (which act on ion transport across membranes) shares several similarities and warrants further pharmacokinetic characterisation. A comprehensive overview of the antiparasitic spectrum of *P. granatum* across target parasites, host species, and experimental models is provided in Table 3.

### 5.4. Antiparasitic Activity Against Fish Monogeneans

Van et al. (2021) [120] evaluated in vitro antiparasitic activity of PPE against *Dactylogyrus* sp., a monogenean gill parasite causing significant mortality and production losses in freshwater aquaculture. PPE at 500 mg/mL achieved 100% cumulative mortality within three minutes of exposure, while 250 and 100 mg/mL achieved equivalent results within six minutes, a rapid and complete parasiticidal effect particularly relevant for bath treatment protocols in aquaculture settings [120]. This represents a potentially transformative application given the limited availability of licensed monogenean treatments and the toxicity of malachite green and other traditional bath compounds.

## 6. Applications in Ruminants, Swine, Aquaculture, and Companion Animals

Pomegranate by-products have been investigated across diverse veterinary applications, encompassing food-producing animals (ruminants, swine, aquaculture species) and companion animals, each presenting distinct physiological contexts and management priorities. While ruminant production systems offer unique opportunities through modulation of rumen fermentation and the valorisation of processing residues within circular economy frameworks, swine and aquaculture applications leverage antimicrobial, antiparasitic, and antioxidant properties, and companion animal applications focus on immunomodulatory and anti-inflammatory benefits.

### 6.1. Ruminants

In cattle, calves supplemented with polyphenols from pomegranate extract showed improved nutrient digestibility, enhanced immune competence (elevated IgG, total protein, lymphocyte counts), and no adverse health effects [121,122]. Unlike monogastric species, ruminants may benefit from the interaction between pomegranate tannins and polyphenols and the rumen microbiota: condensed tannins can bind dietary and microbial proteins, reducing ruminal proteolysis and increasing bypass protein flow to the small intestine, while polyphenols may modulate methanogen populations and biohydrogenation pathways, with potential indirect effects on nutrient utilisation, fermentation efficiency, and enteric methane output [123,124,125,126,127].

In sheep, ethanolic and aqueous macerates of pomegranate peel and leaves demonstrated significant faecal egg count reduction against gastrointestinal nematodes [91,96], consistent with the broad-spectrum anthelmintic activity driven by tannin and alkaloid fractions. In the context of multidrug-resistant *Haemonchus contortus*, the “Barberpole worm”, responsible for the most economically devastating parasitic disease of small ruminants globally, sustainable control strategies that include plant-based options such as pomegranate are increasingly advocated in integrated parasite management programs [92,96].

Pomegranate seed oil and peel extracts added to ram semen extenders significantly improved post-thaw sperm motility, viability, mitochondrial membrane potential, membrane integrity, and reduced MDA concentration, validating practical application in livestock reproductive biotechnology [128,129]. Similar antioxidant protection of bull spermatozoa during cryopreservation has been reported, which has direct implications for the preservation of elite sire genetics [128].

### 6.2. Swine

The relevance of PG alkaloids as anthelmintics for *Ascaris suum* infection in pigs is supported by the in vitro data of Amelia et al. (2017) [82]. Beyond its anthelmintic potential, research on pomegranate in the context of swine colibacillosis highlights a dual therapeutic potential: a direct bactericidal effect, as observed in vitro against multidrug-resistant *E. coli* strains isolated from piglets (average MIC of 5.94 mg/mL for the rind extract), and a clinical in vivo efficacy, where administration of pomegranate-based extracts via drinking water significantly reduced faecal *Enterobacteriaceae* and *E. coli* counts, improved faecal scores (thereby mitigating post-weaning diarrhoea), and enhanced the average daily gain of pigs challenged with the pathogen [130,131]. This aligns with the documented antidiarrheal activity of pomegranate peel aqueous extract has been documented in animal models [132], suggesting its potential relevance for managing diarrhoeal conditions in livestock, including neonatal pigs. Despite these promising findings, it has acknowledged that controlled in vivo studies specifically in swine remain limited, and this represents a significant research gap. Nevertheless, the potential applications of *P. granatum* in swine nutrition extend beyond anthelmintic use.

Notably, the immunomodulatory and antioxidant properties of pomegranate by-products could address oxidative stress associated with intensive swine production systems, which contributes to reduced growth performance and increased disease susceptibility [133].

### 6.3. Aquaculture

Beyond antiparasitic applications against *Dactylogyrus* spp. [120], PPE shows considerable promise as a feed additive and preservative in aquaculture. The antibacterial activity of PPE against fish-pathogenic bacteria, including *Pseudomonas* and *E. coli* strains, provides additional value in disease prevention in intensive aquaculture systems, where antibiotic use is under increasing regulatory pressure [134]. As a feed additive, dietary PPE supplementation in *Carassius auratus* at levels up to 2% significantly reduced enteric Gram-negative bacteria without adversely affecting growth performance, although higher inclusion (4%) impaired growth, likely due to the anti-nutritional effects of tannins [135]. Importantly, PPE at 2% of the diet increased total fungi count, suggesting a selective antimicrobial effect that may benefit gut ecosystem balance [135]. These findings highlight the dose-dependent duality of pomegranate effects, consistent with observations in poultry and other species.

### 6.4. Companion Animals

Clinical evidence for pomegranate applications in companion animals remains sparse but mechanistically grounded, although the majority of the supporting data derive from in vitro studies or non-target species models. The antileishmanial activity of PPE is directly relevant to canine leishmaniasis in endemic regions, where long-term treatment with meglumine antimoniate or miltefosine is required and associated with significant side effects and drug resistance. In vitro evidence supports PPE as an adjunctive or alternative therapy, pending controlled clinical trials in naturally infected dogs [107]. In dogs, the antibacterial activity of pomegranate extracts has been documented against pathogens commonly associated with canine otitis externa and skin infections, conditions frequently complicated by antimicrobial resistance [136]. In a study on clinical isolates from dogs with otitis externa and diarrhoea, pomegranate leaf extract demonstrated significant antibacterial activity against *Staphylococcus* spp. (including coagulase-positive and coagulase-negative strains), *Pseudomonas aeruginosa*, *Proteus* spp., *Klebsiella* spp., and *E. coli*, with minimum bactericidal concentrations (MBC) ranging from 33.33 to 66.66 mg/mL [137]. The extract was particularly effective against multiple drug-resistant isolates, with *Staphylococcus aureus* showing the greatest sensitivity, followed by *Klebsiella*, *E. coli*, and *Salmonella*, while *Proteus* and *Pseudomonas* were more refractory [137]. These findings suggest potential as a topical or systemic adjunctive therapy for canine pyoderma and otitis externa, conditions where antimicrobial resistance is an escalating concern.

Beyond antimicrobial applications, pomegranate polyphenols have demonstrated beneficial effects on canine cardiovascular health. In an in vitro model using canine aortic endothelial cells, pomegranate extract (1–250 μg/mL) significantly inhibited apoptosis and proliferation in a dose-dependent manner, with the highest concentration (250 μg/mL) reducing apoptosis by 73% compared to controls [138]; whether these concentrations are achievable in canine plasma after oral administration is unclear, and it remains to be established whether comparable protective effects occur at physiologically relevant systemic concentrations. This antiapoptotic and antiproliferative activity suggests a vasoprotective effect that could beneficially influence chronic mitral valvular insufficiency (CMVI), the leading cause of heart failure in dogs. The combination of pomegranate extract with soy isoflavones showed comparable effects, indicating potential for nutraceutical interventions in canine cardiovascular disease [138].

The anti-inflammatory and antioxidant properties of pomegranate compounds, including their inhibition of COX-2, NF-κB, and pro-inflammatory cytokines (TNF-α, IL-1β, IL-6, IL-8) are mechanistically consistent with a potential adjunctive role in the management of chronic inflammatory conditions in dogs and cats, including osteoarthritis, inflammatory bowel disease, and skin hypersensitivity disorders, where prolonged NSAID use carries gastrointestinal and renal risk [139,140]. In a clinical study on dogs with keratoconjunctivitis sicca (KCS), a nutraceutical diet containing pomegranate extract (among other botanicals) significantly improved tear production (Schirmer tear test values increased from 4.7 ± 0.4 mm to 10.7 ± 0.6 mm), conjunctival inflammation, corneal keratinisation, and corneal pigment density after 60 days of treatment [141]. These effects were reversed upon diet suspension and restored upon reintroduction, confirming the specific contribution of nutraceuticals to clinical improvement [141]. This suggests a potential role for pomegranate-containing diets in managing immune-mediated ocular diseases in dogs.

In rabbits, which are both production animals and increasingly kept as companion animals, the immunostimulatory effects of pomegranate fruit rind powder have been confirmed. Oral administration of PGFRP at 100 mg/kg for 10 days significantly enhanced both humoral and cell-mediated immune responses in New Zealand White rabbits. Specifically, PGFRP elicited a significant increase in antibody titres against typhoid-H antigen, indicating enhanced responsiveness of macrophages and T and B lymphocyte subsets involved in antibody synthesis. Furthermore, the treatment significantly augmented cell-mediated immunity, as evidenced by increased leucocyte migration inhibition in the Leucocyte Migration Inhibition Test (LMIT) and enhanced skin induration in the delayed-type hypersensitivity (DTH) test with purified protein derivative (PPD) [142]. These findings suggest that pomegranate fruit rind powder possesses appreciable immunostimulatory activity, likely mediated through the enhancement of macrophage, T-cell, and B-cell functions, and support its potential application as an immunomodulatory feed additive across multiple veterinary species.

Furthermore, dietary supplementation of pomegranate peel (3% of diet) and garlic powder (3% of diet) in pregnant does (female rabbits) improved reproductive performance, with the pomegranate peel group showing a 28.5% increase in litter size at birth compared to controls. Birth weight increased by 9.2% in the pomegranate group, 7.2% in the garlic group, and 10.6% in the combination group. Supplementation also enhanced haemoglobin levels in kits, reduced triglycerides, and increased progesterone hormone levels, indicating beneficial effects on reproductive and metabolic health [143]. These results highlight the potential of pomegranate by-products as natural feed additives to improve reproductive efficiency in rabbits.

### 6.5. Laboratory Animals: Models for Veterinary and Comparative Medicine

The rodent model evidence base for *P. granatum* is extensive and provides mechanistic insights applicable to veterinary species. Key findings include: (1) PPE protects against HgCl2-induced oxidative damage in rats by restoring erythrocyte antioxidant capacity and plasma glutathione [144]; (2) pomegranate juice reduces MDA, cholinesterase activity, and lipid peroxidation in the brain of high-fat, high-fructose diet-induced obese rats [145]; (3) PPE administration in diabetic and hypercholesterolaemic rats significantly reduced serum glucose, triglycerides, cholesterol, LDL, urea, uric acid, and creatinine while elevating HDL [146]; and (4) the combination of pomegranate extract and exercise exerts additive anti-inflammatory and immunorestorative effects in diet-induced obese rats [147]. These findings collectively support translational applications in endocrinologically relevant veterinary conditions including equine metabolic syndrome, feline diabetes mellitus, and bovine hepatic lipidosis. The translational applications of pomegranate preparations in ruminants, aquaculture, companion animals, and laboratory models, along with the corresponding key findings, are summarized in Table 4.

## 7. Emerging Pharmacological Activities with Potential Veterinary Relevance

### 7.1. Antimicrobial Activity: Bacteria and Fungi

The antibacterial spectrum of PG extracts is broad, encompassing Gram-positive organisms (*S. aureus* including MRSA, *L. monocytogenes*, *B. cereus*, *C. perfringens*, *E. faecalis*) and Gram-negative organisms (*E. coli*, *Salmonella* spp., *P. aeruginosa*, *K. pneumoniae*, *S. typhimurium*), several of the most clinically significant veterinary bacterial pathogens. Minimum inhibitory concentrations (MICs) documented across studies range from 0.125 mg/mL to 500–1000 µg/mL against less-susceptible Gram-negatives [149,150].

The antibacterial mechanisms involve: (1) precipitation of cell membrane proteins by polyphenols, causing membrane permeabilisation and cell lysis; (2) blockade of microbial enzymes through interaction with sulfhydryl groups; (3) chelation of metal ions required for bacterial enzyme activity; (4) disruption of electron transport and ATP synthesis; and (5) DNA and RNA synthesis inhibition [149,150]. Critically, these multi-target mechanisms reduce the probability of resistance development compared with single-target antibiotics.

Antifungal activity of PPE has been confirmed against *Candida albicans* (MIC 25 µg/mL), *Aspergillus niger,* and *Fusarium oxysporum*, pathogens relevant in avian mycosis, bovine mastitis, and food contamination contexts [151]. Punicalagin demonstrated particularly potent antifungal activity in clinical isolate studies, with MIC values comparable to conventional azoles [152,153]. Notably, the antibacterial activity of pomegranate extracts extends to *Pasteurella haemolytica*, a primary causative agent of pneumonic pasteurellosis (shipping fever) in cattle and sheep, and a pathogen of significant economic importance in livestock production [154]. In a study investigating 504 lung samples from deceased sheep, *P. haemolytica* was isolated from 20.63% of cases and exhibited resistance to all 18 tested antibiotics, including ampicillin, cephalexin, ciprofloxacin, gentamicin, enrofloxacin, and oxytetracycline. Ethanolic extracts of pomegranate pericarp, leaves, flowers, and seeds demonstrated significant antibacterial activity against these multi-drug resistant *P. haemolytica* isolates. The pericarp ethanol extract was the most effective, producing a 24 mm inhibition zone, followed by leaves (14 mm), flowers (12.3 mm), and seeds (9.8 mm). Water and petroleum ether extracts showed no activity, indicating that the active antibacterial compounds are preferentially extracted by ethanol and are likely polar phenolic compounds, including tannins, anthocyanins, flavonoids, and ellagitannins [154]. This finding is particularly significant given the increasing antimicrobial resistance in *P. haemolytica* and the urgent need for alternative therapeutic options in veterinary medicine.

In the context of bovine mastitis, a disease of major economic importance in dairy production, pomegranate flower extracts have demonstrated significant antibacterial activity against mastitis-associated *Staphylococcus* spp. Methanol, ethanol, and water extracts of pomegranate flowers collected from different regions of Turkey were tested against *Staphylococcus aureus* (strains 17 and 18) and six coagulase-negative *Staphylococcus* (CNS) isolates [155]. The methanol extract of pomegranate flowers from Muğla produced the highest inhibition zones, with 22 mm against CNS-37 and 20 mm against S. aureus-18, while the ethanol extract showed 18 mm inhibition zones against S. aureus-17, S. aureus-18, and CNS-36 [155]. The DPPH antioxidant activity of the extracts reached up to 90.1%, indicating a strong correlation between antibacterial and antioxidant activities. The antibacterial mechanism is attributed to phenolic compounds, including gallic acid, ellagic acid, punicalagin, and flavonoids, which can disrupt bacterial cell walls, interfere with membrane-integrated enzymes, alter fatty acid and phospholipid components, and disrupt enzymatic mechanisms for energy production and metabolism [155]. These findings suggest that pomegranate flower extracts could be valuable natural alternatives for the prevention and treatment of bovine mastitis, reducing reliance on antibiotics and mitigating the risk of antimicrobial resistance.

### 7.2. Anti-Inflammatory Activity

Pomegranate polyphenols exert anti-inflammatory effects through multiple pathways, characterised primarily in vitro and in rodent models: inhibition of NF-κB nuclear translocation (suppressing downstream pro-inflammatory gene expression); inhibition of mitogen-activated protein kinase (MAPK) phosphorylation (ERK1/2, p38, JNK); suppression of cyclooxygenase-2 (COX-2) and prostaglandin E2 (PGE2) synthesis; and reduction in pro-inflammatory cytokines (TNF-α, IL-1β, IL-6, IL-8, IL-13) [156,157]. The granatin B compound is proposed as a specific marker of pomegranate anti-inflammatory potency [157]. In broiler chickens with LPS-induced systemic inflammation, PgTc extract reversed TLR overexpression and cytokine storm [158], effects directly relevant to the management of septicaemic and enteric diseases in livestock.

### 7.3. Antidiabetic and Metabolic Effects

The antidiabetic activity of *P. granatum* is mediated, based principally on in vitro enzymatic assays and rodent studies by inhibition of α-glucosidase (IC50 15.18–53.34 U/L), pancreatic lipase (IC50 33.74 µg/mL), and α-amylase (IC50 43.24 µg/mL), as well as activation of PPAR-γ and the insulin signalling pathway (Akt/GSK-3β phosphorylation) and suppression of the ER stress marker IRE1α-XBP1 [10,159,160]. Gallic acid, oleanolic acid, ellagic acid, ursolic acid, and punicalagin are the principal antidiabetic bioactives. These mechanisms are directly relevant to managing feline diabetes mellitus, equine metabolic syndrome, and bovine ketosis, conditions characterised by insulin resistance, oxidative stress, and lipid peroxidation that are exact targets of PG’s pharmacology.

In the context of metabolic syndrome (MetS), a condition increasingly recognized in veterinary species (equine metabolic syndrome, feline diabetes mellitus, canine obesity-related disorders), pomegranate peel extract has demonstrated several beneficial effects. In a study using Zucker Diabetic Fatty rats (ZDF, fa/fa), an established animal model for MetS, supplementation with pomegranate peel extract at 100 and 200 mg/kg for eight weeks was found to reduce oxidative stress in kidney tissue. This was evidenced by decreased levels of ROS/RNS, oxLDL, and SOD activity, alongside increased CAT activity and a trend toward lower kidney injury markers (KIM-1) compared to untreated MetS controls [161].

In hypercholesterolemic rats, dietary supplementation with pomegranate peel powder (10%, 15%, and 20% of diet) significantly reduced serum total cholesterol, triglycerides, LDL, and VLDL levels compared to hypercholesterolemic controls, while maintaining or increasing HDL levels. Total cholesterol decreased from 146.05 ± 3.91 mg/dL in hypercholesterolemic controls to 89.54 ± 4.12 mg/dL (10% PPP), 92.03 ± 3.64 mg/dL (15% PPP), and 95.32 ± 3.37 mg/dL (20% PPP). The hypocholesterolemic effect is attributed to the high polyphenolic content of pomegranate peel, particularly ellagic acid and tannic acid, which possess potent antioxidant activity and can reduce lipid peroxidation [162]. Additionally, pomegranate polyphenols may enhance paraoxonase-1 (PON-1) activity, an HDL-associated enzyme that protects against lipid peroxidation and is inversely correlated with cholesterol levels and atherosclerosis risk [163,164]. The hypolipidemic effects of pomegranate are further supported by studies demonstrating that pomegranate peel polyphenols upregulate liver X receptor α (LXRα), PPARα, and PPARγ, and downregulate fatty acid synthase through inhibition of keto-acetyl synthase and acetyl/malonyl transferase domains, promoting cholesterol removal via enhanced faecal bile acid excretion [165,166].

### 7.4. Hepatoprotective and Nephroprotective Activity

Pomegranate peel extract has been shown to reduce oxidative damage and improve tissue structure in two different rat models of liver injury. In a model of biliary obstruction, Toklu et al. demonstrated that pomegranate peel extract reduced oxidative stress (lowered MDA and MPO), increased antioxidant capacity (GSH), and improved hepatic structure and function [167]. Similarly, in a CCl4-induced hepatotoxicity model, Wei et al. (2019) reported that pomegranate peel extract decreased MDA levels, enhanced GSH-Px activity, and inhibited liver fibrosis through the p38MAPK/Nrf2 pathway [168]. These hepatoprotective effects translate directly to veterinary applications in bovine liver disease, toxic hepatopathies in small animals, and periparturient dairy cow liver mobilisation disorders. Nephroprotective effects have been documented in gentamicin-induced nephropathy models, indicating potential utility in monitoring renal function during antibiotic therapy in small animals.

The nephroprotective potential of pomegranate has been demonstrated in models of acute renal failure. In Wistar rats with gentamicin-induced nephrotoxicity (80 mg/kg i.p. for 7 days), administration of pomegranate fruit extract (1 mL orally) helped maintain serum total protein and modulated uric acid levels, although the effects on other biochemical and haematological parameters were moderate and dose-dependent [169]. The protective effect is mediated through the potent antioxidant activity of pomegranate polyphenols, particularly punicalagin and ellagic acid, which scavenge reactive oxygen species generated by gentamicin, prevent lipid peroxidation, and preserve the structural and functional integrity of renal tubular epithelial cells [170]. These findings suggest that pomegranate extracts could be valuable in managing drug-induced nephrotoxicity in veterinary patients, particularly when aminoglycoside antibiotics are used in the treatment of severe infections.

### 7.5. Wound Healing

*P. granatum* extracts significantly accelerate wound healing by reducing ulcer size, increasing wound contraction rate and breaking strength, stimulating fibroblast migration (enhanced by punicalagin + Zn (II) combination), and upregulating FGF-2 and TGF expression in socket tissue [171]. These effects support clinical applications in wound management in veterinary species, particularly important in farm animals where chronic wounds, digital dermatitis, and foot rot impose substantial welfare and economic burdens.

### 7.6. Immunomodulatory Activity

The immunomodulatory properties of *P. granatum* have been demonstrated across multiple species. In rabbits, oral administration of pomegranate fruit rind powder (100 mg/kg) significantly enhanced both humoral and cell-mediated immune responses [142]. The treatment increased antibody titres against typhoid-H antigen in the Widal tube agglutination test and augmented cell-mediated immunity as evidenced by increased leucocyte migration inhibition and enhanced delayed-type hypersensitivity reactions to PPD. These effects are attributed to enhanced macrophage, T-cell, and B-cell functions, with macrophages playing a pivotal role in antigen processing and presentation to T and B lymphocytes [142].

In rabbits, dietary supplementation with pomegranate peel (1.5%) in combination with garlic powder (1.5%) significantly increased immunoglobulin G (IgG) levels, although immunoglobulin M (IgM) levels were decreased. The combination treatment showed a 66.19% and 33.46% increase in IgG compared to pomegranate alone and garlic alone, respectively [143]. These findings suggest that pomegranate polyphenols can modulate immunoglobulin production, potentially through the activation of lymphocytes and macrophages and the regulation of gene expression for cytokines, antibodies, and cytotoxic substances.

The immunostimulatory effects of pomegranate are likely mediated through multiple mechanisms: (1) activation of macrophages and enhancement of antigen processing and presentation [172]; (2) stimulation of T-lymphocyte proliferation and differentiation [173]; (3) modulation of cytokine production (TNF-α, IL-6, IFN-γ); and (4) enhancement of antibody synthesis by B lymphocytes [172]. These properties make pomegranate a promising natural immunomodulator with potential applications in enhancing vaccine responses, managing infectious diseases, and improving overall immune competence in veterinary species.

### 7.7. Antigenotoxic and Antimutagenic Activity

The genoprotective potential of pomegranate peel extract has been investigated using in vivo models. In a study on mice, pomegranate peel extract administered at 150 and 300 mg/kg for 15 days significantly mitigated the genotoxic effects induced by mitomycin-C (MMC), a potent alkylating agent that causes DNA cross-linking and chromosomal aberrations. MMC treatment increased chromosomal aberration rates (including chromosome fractures, fragments, sister chromatid associations, and chromatid fractures) and micronucleated polychromatic erythrocyte (MNPCE) frequencies while decreasing mitotic activity and the PCE/NCE ratio. Pomegranate peel extract co-administration significantly reduced chromosomal aberrations and micronucleus formation in a dose-dependent manner (*p* < 0.001), restored mitotic activity, and normalized the PCE/NCE ratio. The extract alone showed no genotoxic effects, with parameters comparable to the control group. Overall, these findings demonstrate that pomegranate peel extract exerts significant antigenotoxic effects against MMC-induced genotoxicity in mouse bone marrow cells, without exhibiting any genotoxic activity on its own, suggesting its potential as a safe natural antigenotoxic agent [174].

These antigenotoxic properties have significant veterinary implications, particularly in the context of (1) mitigating the genotoxic side effects of chemotherapeutic agents used in veterinary oncology; (2) protecting livestock and companion animals from environmental genotoxins (pesticides, mycotoxins, heavy metals); and (3) preserving genetic integrity in breeding animals.

### 7.8. Reproductive and Fertility-Enhancing Effects

The antioxidant and hormone-modulating properties of *P. granatum* have demonstrated significant potential for improving reproductive performance across multiple species.

In male reproductive health, pomegranate extract has shown protective effects against heat-induced testicular damage. In a study on albino Wistar rats exposed to heat stress (40–42 °C for 15 min daily for 14 days), administration of a standardized 40% ellagic acid pomegranate extract at 300 mg/kg/day significantly increased seminiferous tubule diameter (335.93 ± 25.85 μm) and epithelial thickness (95.9 ± 8.49 μm) compared to heat-exposed controls (221.43 ± 10.50 μm and 78.11 ± 14.24 μm, respectively). The protective effect is attributed to the antioxidant activity of pomegranate polyphenols, particularly anthocyanins, ellagic acid, and punicalagin, which neutralize free radicals generated by heat stress, preventing oxidative damage to Sertoli cells and spermatogonia, and preserving normal spermatogenesis. The dominant antioxidant compound anthocyanin may also influence hormonal regulation of FSH, LH, and testosterone, affecting the growth and development of Sertoli cells and spermatogonia [175].

In female reproductive health, dietary supplementation with pomegranate peel (3% of diet) in pregnant rabbits significantly improved reproductive performance, increasing litter size at birth by 28.5% compared to controls, with birth weights increased by 9.2%. Serum progesterone levels were significantly elevated in the pomegranate-treated group (increased by 34.6% compared to controls). The improvement in reproductive efficiency is likely mediated through the antioxidant effects of pomegranate polyphenols, which protect against lipid peroxidation in cell membranes and through the modulation of gonadotropin secretion and ovarian hormone production [143].

Additionally, pomegranate has demonstrated protective effects against testicular toxicity induced by xenobiotics. In a study on carbon tetrachloride-induced testicular damage in rats, pomegranate juice significantly reduced oxidative stress and preserved testicular architecture [176]. These findings collectively suggest that pomegranate extracts and by-products could be valuable natural supplements for enhancing fertility and reproductive performance in both male and female animals, with applications in livestock breeding, companion animal reproduction, and the management of reproductive disorders.

## 8. Safety, Toxicology, and Drug Interactions

The safety profile of *P. granatum* preparations is generally favourable, with extensive traditional use and a growing body of toxicological evidence supporting their safety at physiological doses [10,177]. Acute oral toxicity studies have reported an LD50 value of 731.1 mg/kg body weight for pomegranate peel powder [10]. In a separate acute toxicity evaluation, the oral LD_50_ of a standardized pomegranate fruit extract in rats and mice was found to be greater than 5 g/kg body weight, indicating that the extract is practically non-toxic, while the intraperitoneal LD_50_ in rats and mice was determined as 217 and 187 mg/kg body weight, respectively [177]. In subchronic toxicity studies, a NOAEL (No-Observed-Adverse-Effect-Level) of 600 mg/kg/day was established for standardized pomegranate peel extract in Wistar rats following 90-day oral administration; at this dose, no treatment-related adverse effects were observed in clinical observations, body weights, feed consumption, haematology, serum chemistry, organ weights, or histopathology [177]. Additional toxicological evaluations have demonstrated no observable toxic effects in BALB/c mice at doses up to 2000 mg/kg for pomegranate peel galactomannan polysaccharides. However, higher concentrations have been found to be increasingly toxic in brine shrimp assays, indicating that dosage must be carefully considered [10]. In experimental broilers, administration of crude pomegranate peel extracts has been investigated for both efficacy and safety; one study reported that supplementation of 400 ppm pomegranate peel extract can induce injuries in the liver, while lower doses had no effect on liver tissue, underscoring the importance of dose optimisation for veterinary applications [6]. Regarding dietary inclusion, research has shown that replacement of 8% pomegranate peel in broiler diets decreases growth performance and reduces apparent nutrient digestibility, with high levels of dietary pomegranate peel powder (8%) associated with decreased intestinal crypt depth and reduced villi/crypts ratio, effects attributed to tannins binding to digestive enzymes such as trypsin, lipase, and amylase, thereby reducing nutrient digestibility [58]. These findings suggest that while lower inclusion levels may offer benefits, high dietary incorporation of pomegranate peel in poultry can have anti-nutritional consequences.

Potential drug interactions represent an important consideration for the clinical use of pomegranate preparations. Pomegranate juice has been shown to be a potent inhibitor of human cytochromes P450, particularly CYP2C9 and CYP3A4; in vitro and animal pharmacokinetic data support the possibility of CYP3A4/CYP2C9 inhibition by pomegranate juice [178]. Specifically, pomegranate juice has been shown to consistently inhibit intestinal CYP2C9 and CYP3A4 enzymes in vitro and in vivo, resulting in increased exposure to drugs metabolised by these pathways; however, the human relevance for clinically significant drug–drug interactions has not been firmly established based on limited case studies [178]. Importantly, CYP isoform expression, substrate specificity, and catalytic activity differ substantially across veterinary species (e.g., dogs, cats, horses), and species-specific pharmacokinetic and drug-interaction data for pomegranate preparations are currently lacking; therefore, the human-derived data should be extrapolated to veterinary patients with considerable caution.

Beyond toxicological considerations, the bioavailability of pomegranate compounds represents a critical factor influencing their clinical efficacy. Pomegranate ellagitannins, such as punicalagin, are not absorbed intact into the blood stream but are hydrolysed in the gastrointestinal tract and metabolised by gut microbiota into urolithins, which are then conjugated in the liver and excreted in the urine (Pantiora et al., 2023) [179]. These urolithin metabolites have demonstrated potent anti-inflammatory, antioxidative, and anticancer effects; they have improved bioavailability and can stay in circulation for a longer time when compared to the parent compound, contributing to the health benefits observed for punicalagin [179]. This microbiota-dependent biotransformation introduces inter-individual and inter-species variability that must be considered in the standardisation of treatments [179].

Nanotechnology-based strategies may help overcome these bioavailability limitations. Recent advances in the synthesis and characterisation of various nanoparticles from pomegranate peel extract (PPE) have been reported [34]. Pomegranate peel extract, rich in bioactive compounds with antibacterial, antioxidant, and anti-inflammatory properties, has been explored for micro- and nanoencapsulation in food systems. Bioavailability of punicalagin has been improved using chitosan nanoparticles and lipid-based self-microemulsifying drug delivery systems (SMEDDS), a lipid-based nanoemulsion that enhances lymphatic transport and bypasses first-pass metabolism [34]. From a translational perspective, these advanced formulations may be particularly valuable for optimising delivery to target tissues; however, the translation to novel, more bioavailable formulations necessitates rigorous, target-specific pharmacokinetic and safety evaluations before widespread clinical adoption [34].

## 9. Translational Perspectives and Research Priorities

Despite the remarkable breadth and depth of evidence surveyed in this review, significant translational gaps limit the immediate clinical application of *P. granatum* in veterinary practice:

**1. Standardisation of preparations.** The phytochemical composition of commercially available PPP, PPE, and PSO varies enormously depending on cultivar, agricultural practices, post-harvest handling, extraction solvent and method, and storage conditions [34,180]. A standardised veterinary-grade pomegranate extract with guaranteed minimum punicalagin and ellagic acid content is needed for reproducible and repeatable clinical outcomes.

**2. Target-species pharmacokinetics.** Bioavailability and gut-mediated biotransformation of ellagitannins to urolithins varies significantly between species based on intestinal microbiota composition. Pharmacokinetic profiling in ruminants (with their complex forestomach fermentation), monogastric species, and carnivores is essential before effective dose regimens can be established [181,182,183].

**3. Controlled clinical trials.** Most of the evidence is preclinical (in vitro or rodent models). Randomised, blinded, controlled clinical trials in naturally infected or production-challenged target species (e.g., Eimeria-challenged broilers, *Haemonchus*-infected sheep, Leishmania-infected dogs) are critically needed to validate efficacy, establish minimum effective doses, and characterise safety margins [34,184].

**4. Formulation innovation.** Nano-encapsulation of pomegranate polyphenols has been shown to significantly improve bioavailability and sustained release kinetics. Nano-encapsulated PPE demonstrated significantly larger inhibitory zones against tested bacteria than conventional PPE. Nano-formulation strategies specifically adapted for veterinary oral and topical delivery represent a major opportunity [185,186].

**5. Combination strategies.** Evidence for synergistic effects with conventional drugs (e.g., pomegranate combined with sodium hypochlorite for *Enterococcus faecalis* biofilm [187]) and with other phytobiotics (e.g., green tea + pomegranate [188]) suggests that combination strategies merit systematic investigation to optimise efficacy and reduce individual compound doses, minimising toxicity risk.

**6. Aquaculture applications.** The aquaculture sector offers perhaps the highest unmet need for safe, ecologically compatible antimicrobials and antiparasitics. The demonstrated antiparasitic, antibacterial, and antioxidant-preservative properties of PPE, combined with its biodegradability and non-accumulation in aquatic ecosystems, position it as a priority candidate for this underexplored application space [189].

**7. Dose translation and practical considerations.** A critical translational gap lies in the translation of experimental doses to practical veterinary applications. The doses effective in preclinical studies, often expressed as mg/kg body weight or as a percentage of diet, may not be directly achievable or economically viable under commercial farming conditions. For example, the 400-ppm PPE dose effective against *Eimeria* in broilers translates to 400 g per tonne of feed, which is economically feasible. However, the 3 g/kg body weight dose of aqueous peel extract used in murine *Cryptosporidium* models would be impractical for large-scale livestock application. Furthermore, doses that are effective in controlled experimental settings may not account for interactions with other dietary components (e.g., phytate, fibre, minerals) that reduce polyphenol bioavailability, nor for the anti-nutritional effects of tannins at higher inclusion levels [180]. The absence of dose–response studies in target species complicates the establishment of safe and effective ranges. A pragmatic approach would involve: (1) conducting stepwise dose-titration studies in the target species under commercial conditions; (2) establishing maximum safe inclusion levels based on growth performance, feed intake, and absence of adverse effects; (3) validating efficacy at the lowest effective dose to maximise cost-effectiveness; and (4) developing standardised extracts with guaranteed bioactive content to enable reproducible dosing. Until such data are generated, practical recommendations must remain conservative, emphasising the importance of species-specific dose optimisation and ongoing monitoring for adverse effects.

## 10. Limitations and Critical Appraisal of the Current Evidence

Despite the remarkable breadth of evidence consolidated in this review, several critical limitations must be acknowledged to contextualise the findings appropriately and guide future research priorities.

### 10.1. Heterogeneity of Pomegranate Preparations

One of the most significant barriers to evidence synthesis is the profound heterogeneity in pomegranate preparations used across studies. Extracts vary enormously in terms of plant part (peel, juice, seed oil, leaves, flowers), cultivar, geographic origin, extraction solvent (aqueous, methanol, ethanol, acetone, supercritical CO_2_), extraction method (maceration, Soxhlet, ultrasound-assisted, microwave-assisted), and degree of standardisation. For example, the punicalagin content of pomegranate peel extracts demonstrates considerable variability across different processing methods. While crude extraction may yield punicalagin at concentrations around 10%, purification using preparative HPLC can achieve purities of 92–98%, and semi-preparative HPLC has been reported to yield punicalagin with a purity exceeding 99% [31]. This variability means that positive findings with one preparation cannot be automatically generalised to others, and negative findings may reflect suboptimal extraction rather than genuine lack of activity. The absence of a universally accepted veterinary-grade standardised extract severely limits reproducibility and clinical translation.

### 10.2. Preclinical Predominance and Species Extrapolation

The vast majority of evidence reviewed remains preclinical, with in vitro studies and rodent models disproportionately represented relative to target veterinary species. While rodent models provide valuable mechanistic insights, they cannot fully recapitulate the complex pathophysiology of diseases in production animals (e.g., poultry, ruminants, swine) or companion animals. Rodent metabolism of ellagitannins to urolithins is not identical to that of other species, and the bioavailability of pomegranate polyphenols varies substantially with gastrointestinal anatomy, transit time, and microbiota composition [181,190,191]. Consequently, doses effective in mice may be either ineffective or toxic in target species, and vice versa.

Furthermore, the number of well-designed randomised controlled trials in clinically affected target species remains strikingly low. Most in vivo veterinary studies involve small sample sizes (often *n* < 20 per group), lack blinding, and fail to report power calculations or standardised outcome measures. This limits the generalisability of findings and increases the risk of Type I and Type II errors. Nonetheless, a recent researcher-blinded randomised controlled trial in dogs showed that a pomegranate-containing water additive significantly reduced plaque and calculus accumulation and improved gingival health after professional dental cleaning, providing an important proof-of-concept that some pomegranate-based formulations can progress beyond the preclinical stage in a target veterinary species [192].

### 10.3. Publication Bias and Positive Outcome Reporting

There is a discernible tendency in the published literature towards reporting positive outcomes, with negative or equivocal results less frequently published (publication bias). This is particularly evident in the poultry sector, where studies reporting improvements in growth performance and antioxidant status are overrepresented, while studies showing no effect or adverse outcomes at higher doses are underrepresented. The dose-dependent duality of pomegranate effects, beneficial at low-to-moderate inclusion levels but detrimental at high levels due to anti-nutritional tannin effects, is acknowledged but may be underreported [193]. Additionally, the lack of registered protocols or pre-registered outcomes for most studies makes it difficult to assess the risk of selective outcome reporting.

### 10.4. Absence of Pharmacokinetic Data in Target Species

Critically, no comprehensive pharmacokinetic studies have been conducted in any veterinary target species. Data on absorption, distribution, metabolism, and excretion (ADME) of punicalagin, ellagic acid, and urolithins in poultry, ruminants, swine, or dogs are almost entirely absent. Without such data, rational dose selection is impossible. The assumption that oral administration of raw pomegranate peel powder delivers the same bioactive concentrations as purified extracts is untested and likely incorrect. This gap is particularly concerning given the extensive gut-microbiota-mediated biotransformation of ellagitannins to urolithins, which is species-dependent and may yield highly variable systemic exposure [171].

### 10.5. Safety Data and Toxicological Gaps

Although the safety profile of pomegranate preparations is generally favourable, systematic toxicological assessments are lacking. Chronic toxicity studies, reproductive toxicity studies, and carcinogenicity studies have not been performed in any veterinary species. The potential for cumulative toxicity from long-term dietary inclusion, interactions with other feed additives, and effects on food safety (residues in meat, milk, and eggs) remain unexplored. The limited genotoxicity data available are reassuring but not definitive [174].

### 10.6. Economic and Practical Constraints

**Figure 3:**
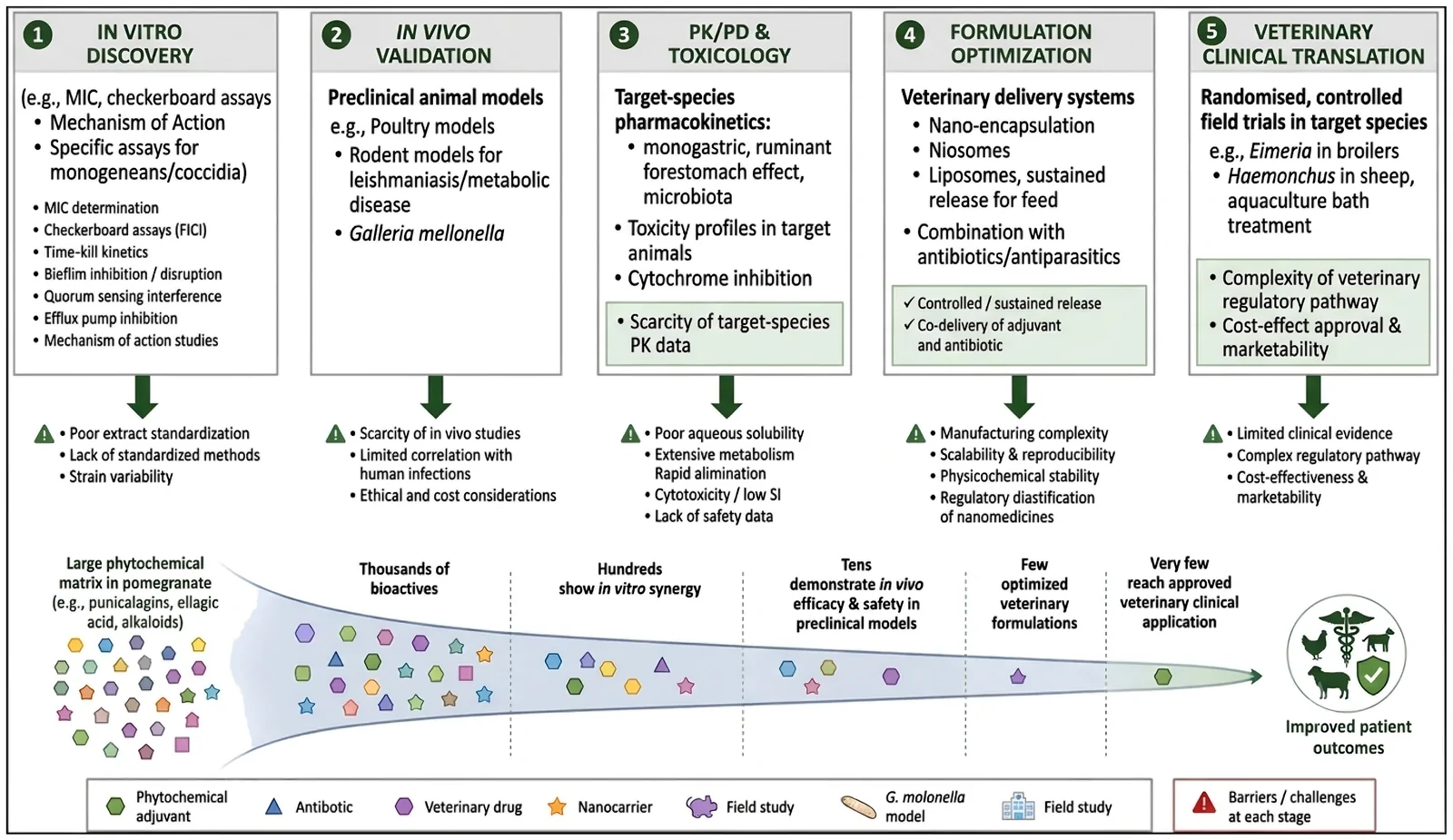
Veterinary Translational Pipeline and Phytocomplex Funnel. This diagram synthesizes the overall conclusions and future research priorities of the review. It illustrates the developmental pathway of pomegranate-derived compounds from initial in vitro discovery to species-specific challenges (e.g., the rumen bypass effect in ruminants) and optimized veterinary formulations, culminating in targeted clinical translation across animal species (e.g., *Eimeria* studies in broilers, *Haemonchus* in sheep, *Leishmania* in dogs, and specialized treatments in aquaculture). The funnel model highlights how the vast and diverse phytochemical matrix of pomegranate is systematically narrowed down toward practical applications by identifying the critical veterinary-specific barriers at each developmental phase.

Finally, many studies are conducted under controlled experimental conditions that do not reflect commercial production realities. The cost-effectiveness of pomegranate supplementation, the logistics of sourcing and processing peel at scale, palatability issues, and the stability of bioactive compounds during feed manufacturing (extrusion, pelleting, storage) are rarely addressed. These factors are critical determinants of whether promising research findings can translate into practical veterinary applications [194]. The translational pipeline from preclinical discovery to clinical veterinary application, including the species-specific barriers and formulation challenges that must be addressed at each developmental phase, is conceptualized in Figure 3.

In summary, while the evidence base for *P. granatum* in veterinary medicine is extensive and promising, it is characterised by significant heterogeneity, a predominance of preclinical data, publication bias, and critical gaps in pharmacokinetics, toxicology, and real-world feasibility. These limitations must be addressed through rigorously designed, adequately powered, and transparently reported studies before pomegranate products can be confidently recommended for routine veterinary use.

## 11. Conclusions

*Punica granatum* L. represents one of the most pharmacologically versatile and scientifically validated phytobiotics available to veterinary medicine. Its rich matrix of punicalagins, ellagic acid, anthocyanins, flavonoids, alkaloids, and punicic acid confers a spectrum of biological activities, antiparasitic, antimicrobial, antioxidant, immunomodulatory, antidiabetic, hepatoprotective, and wound-healing, that address some of the most pressing challenges facing contemporary animal health: antimicrobial resistance, antiparasitic drug resistance, oxidative stress in intensive production systems, and metabolic disease.

In poultry—the species group with the most extensive evidence base—pomegranate peel products at 0.01–1.5% dietary inclusion consistently improve production performance, antioxidant status, and humoral immunity, and exert meaningful anticoccidial activity. In ruminants, antiparasitic applications against gastrointestinal nematodes and protection of semen quality during cryopreservation are well-supported. In aquaculture, rapid and complete parasiticidal activity against gill monogeneans and potent antimicrobial and antioxidant preservative properties are documented. Translational potential in companion animal medicine for leishmaniasis, inflammatory disease, and wound management is scientifically credible and deserves clinical exploration.

The safety profile of appropriately dosed pomegranate preparations is generally favourable, with dose-dependent pro-tannin anti-nutritional effects at high dietary inclusion being the primary constraint in livestock applications. Standardisation of extract quality, species-specific pharmacokinetic characterisation, and controlled clinical trials in target species are the foundational next steps required to realise the full veterinary potential of this ancient, globally available, and economically accessible medicinal plant.

## Figures and Tables

**Table 1:** Principal phytochemical constituents of the different parts of *Punica granatum* L. and their principal pharmacological activities in veterinary contexts.

Plant Part	Major Phytochemicals	Key Biological Functions	References
Fruit peel/rind	Punicalagin A & B, punicalin, ellagic acid, gallic acid, ellagitannins, gallotannins, flavonoids (catechin, epicatechin), anthocyanins, condensed tannins, alkaloids (pelletierine), phenolic acids	Antioxidant, antimicrobial, antiparasitic, anti-inflammatory, antifungal, anthelmintic, anticoccidial	[9,13]
Fruit juice	Anthocyanins, ellagic acid, catechins, quercetin, rutin, gallic acid, punicalagin, caffeic acid, proanthocyanidins, organic acids, vitamins C & K	Cardiovascular protection, antioxidant, antidiabetic, anti-atherosclerotic, immunomodulatory	[9]
Seeds/seed oil	Punicic acid (conjugated linolenic acid), linoleic acid, oleic acid, palmitic acid, stearic acid, tocopherols, sterols, phytoestrogens, ellagic acid, lignins	Lipid profile improvement, immunomodulation, anti-obesity, reproductive performance enhancement	[9,10]
Leaves	Punicalin, punicalagin, punicafolins, tannins, apigenin, naringin, flavone glycosides, anthocyanins, saponins, alkaloids, minerals (K, Ca, Fe, Mg, Zn)	Antidiabetic, anthelmintic, antibacterial, antidandruff, anti-obesity, antioxidant	[9,13]
Flowers	Gallic acid, ursolic acid, maslinic acid, oleanolic acid, terpenic compounds, flavonoids, tannins, catechin, anthocyanins	Antidiabetic, anti-inflammatory, antibacterial (mastitis pathogens), uterine activity	[9,13]
Roots/bark	Piperidine alkaloids (pelletierine, pseudopelletierine, methylpelletierine), ellagitannins, tannins	Anthelmintic (taeniacidal/vermifugal), antiprotozoal	[9,13]

**Table 2:** Summary of the principal effects of *Punica granatum* supplementation on production, antioxidant, immune, and metabolic parameters in poultry species.

Species	PG Product/Dose	Main Outcomes	Antioxidant/Immune	Blood/Lipids	Reference
Broiler chicken	PPP 0.25–1.50%	Improved BW, BWG, FCR; reduced abdominal fat; increased lymphoid organ weight	↑ IgA, IgG, SOD, GPx; ↓ MDA in breast meat	↓ Cholesterol, LDL; ↓ ALT, AST	[51]
Broiler chicken	PPE 250–650 mg/kg	Improved carcass quality; increased water-holding capacity during storage	↑ IgG, anti-SRBC titres; ↓ breast-muscle MDA	↓ Cholesterol, LDL; ↑ HDL	[80]
Broiler chicken	PPP 7.5–10 g/kg	↓ Heart-to-body weight ratio; protective against pulmonary hypertension	↑ Hepatic CAT & SOD; ↑ NO	↓ TG, cholesterol, abdominal fat deposition	[68]
Broiler chicken	PPP 3 g/kg and 6 g/kg	↓ Cecal lesion scores (*p* < 0.05) at both doses; ↓ Oocyst shedding at 5, 7, 9 DPI (*p* < 0.05); ↑ Weight gain and ↓ FCR (*p* < 0.05) at 6 g/kg; Restoration of cecal villus height, crypt depth, and VH:CD ratio; ↓ Mortality at both doses	Polyphenolic compounds (punicalagin, ellagitannins, anthocyanins) exert antiprotozoal, anti-inflammatory, and antioxidant activities; ↑ beneficial microbiota; Protection of intestinal epithelial cells from oxidative damage	No hepatotoxicity or adverse effects reported at tested doses	[74]
Layer hen	PPP 4–5%	↑ Egg production; ↓ oxidative stress in yolk	↑ SOD, GPx; ↓ MDA; ↑ total antioxidant capacity	↓ Serum cholesterol	[77]
Japanese quail	PPP 4.5–7.5%	↑ Egg production, egg weight, egg mass	↑ Antioxidant activity; ↑ immune cell count	↓ Lipid peroxidation	[79]

↑ symbol indicates an increase; ↓ symbol indicates a decrease.

**Table 3:** Summary of experimental evidence supporting the antiparasitic activity of *Punica granatum* across parasite taxa and host species.

Target Parasite	Host/Model	PG Part/Dose	Efficacy	Reference
*Hymenolepis nana*	Swiss albino mice	PPE 300 mg/kg	↓ Egg excretion; reduction in worm burden	[86]
*Schistosoma mansoni*	Mice (in vitro & in vivo)	Peel/leaf extract 800 mg/kg × 45 days	100% worm mortality in vitro; 72–77% adult worm death in vivo	[89]
*Giardia lamblia*	Mice	PPE (oral, 28 days)	97.4% cure rate by day 28; ↓ trophozoites & cysts	[119]
*Plasmodium falciparum*	In vitro; murine model	Ellagic acid, punicalagin; peel methanolic extract IC50 6.7 µg/mL	Growth inhibition via oxidative stress induction; ↓ spleen injury	[104]
*Cryptosporidium parvum*	Neonatal albino mice	PPE aqueous, 3 mg/kg	Full elimination of oocyst shedding; ↑ BW; ↓ intestinal histopathology	[103]
*Eimeria tenella*	Broiler chickens	PPP 3 g/kg and 6 g/kg in feed	↓ Cecal lesion scores (*p* < 0.05) at both doses vs. PC; ↓ Oocyst shedding (OPG) at 5, 7, 9 days post-infection (*p* < 0.05); ↑ Weight gain and ↓ FCR at 6 g/kg (*p* < 0.05); ↓ Mortality; Restoration of cecal villus height, crypt depth, and VH:CD ratio; Comparable efficacy to amprolium (1 g/kg) at 6 g/kg for most parameters	[74]
*Ascaris suum*	Female A. suum (in vitro)	PPE 75%	82% worm mortality; concentration-dependent effect	[82]
*Dactylogyrus* sp.	Fish (in vitro)	PPE 500 mg/mL	100% cumulative mortality at 3 min; concentration-dependent	[120]
*Haemonchus/Cooperia* spp.	Cattle (larval inhibition assay)	Pomegranate stem bark aqueous extract	Significant in vitro larval inhibition	[97]
*Leishmania infantum*	In vitro promastigotes	PPE (NMR/HPLC characterised)	Dose-dependent growth suppression; apoptosis-mediated leishmanicidal effect	[107]

↑ symbol indicates an increase; ↓ symbol indicates a decrease.

**Table 4:** *Punica granatum* applications in ruminants, aquaculture, companion animals, and rodent models: key findings and translational relevance.

Species	PG Product/Dose	Condition/Endpoint	Key Findings	Reference
*Cattle* (*calves*)	PG extract (polyphenols)	Growth, immunity, gut health	Improved nutrient digestibility and immune competence; no adverse effects on health	[121]
*Sheep* (*lambs/does*)	PPE ethanolic, aqueous macerates	Gastrointestinal nematodes (egg count reduction)	50% anthelmintic effect; significant faecal egg count reduction	[96]
*Cattle* (*bovine worms*)	Pomegranate stem bark extract	*Haemonchus/Cooperia* inhibition assay	In vitro larval inhibition comparable to reference anthelmintic	[92]
*Ram/Bull semen*	PPE added to semen extender (variable concentrations)	Post-thaw sperm quality	↑ Motility, viability, membrane integrity, mitochondrial activity; ↓ MDA (lipid peroxidation)	[128]
*Rat/rodent models*	PG 200 mg/kg/day	Streptozotocin-induced diabetes; obesity; hepatotoxicity	↓ Blood glucose, cholesterol, LDL, TG, ALT, AST; ↑ HDL; hepatoprotective effect	[148]

↑ symbol indicates an increase; ↓ symbol indicates a decrease.

## Data Availability

No new data were created or analyzed in this study. Data sharing is not applicable to this article.
